# Correlates of Maximal Driver Club Head Speed in Elite Male and Female Golfers: The Role of Maximal Muscle Strength, Power, and Anthropometry

**DOI:** 10.1111/sms.70255

**Published:** 2026-03-12

**Authors:** M. J. Johansen, P. Aagaard, C. Bishop, T. Kvorning, K. D. Gejl, J. Bojsen‐Møller

**Affiliations:** ^1^ Department of Sports Science and Clinical Biomechanics University of Southern Denmark Odense Denmark; ^2^ Faculty of Science and Technology, London Sport Institute Middlesex University London UK

**Keywords:** club head speed, countermovement jump, golf performance, isometric strength, muscle power, muscle strength

## Abstract

This study examined associations between driver club head speed (dCHS) and strength, power, and anthropometric measures in elite golfers, analyzed in males and females. Forty‐one golfers (22 males, 19 females), including PGA and LPGA Tour professionals, completed a standardized test battery comprising golf swing testing (TrackMan launch monitor), countermovement jump (CMJ), isometric mid‐thigh pull (IMTP), isometric bench press (IBP), and trunk rotation power testing. In males, dCHS showed very strong associations with trunk rotation peak power (*r* = 0.89, 95% confidence intervals [0.72; 0.96]), CMJ impulse and peak power (*r* = 0.78 [0.53; 0.90]), and IMTP peak force (*r* = 0.75 [0.47; 0.90]), and a strong association with IBP peak force (*r* = 0.68 [0.35; 0.86]). In females, dCHS correlated strongly with CMJ impulse (*r* = 0.67 [0.30; 0.87]), CMJ peak force (*r* = 0.66 [0.28; 0.86]), IBP peak force (*r* = 0.60 [0.18; 0.83]), and trunk rotation peak power (*r* = 0.59 [0.16; 0.82]). Median‐split analyses confirmed that high‐dCHS golfers consistently outperformed those with lower‐dCHS across key strength‐ and power‐related measures, with anthropometric variables further differentiating high‐ from low‐dCHS females. These findings highlight both shared and sex‐specific associations of dCHS with physical performance in elite golfers and support the use of CMJ, IMTP, IBP, and trunk rotation power testing for profiling and longitudinal monitoring in this population.

## 1 Introduction

Driving distance is a critical determinant of elite golf performance, with golfers who generate greater distance consistently outperforming those with shorter total driving distance across different competitive levels [1, 2, 3]. Improvements in driving distance are directly associated with lower scoring averages in elite golfers, with PGA Tour data showing that driving distance contributes significantly to better scoring on par 4 and 5 holes [4]. Further, more recent European Tour data indicate that distance also impacts performance on par 3 holes, likely because golfers with greater distance can approach the green with shorter clubs and thereby achieve higher accuracy [5]. Club head speed (CHS) is the primary swing‐related determinant of ball release speed and thereby driving distance [6, 7, 8]. Thus, CHS is a key determinant of golf performance and warrants investigation of its relationships with mechanical muscle function characteristics, including maximal force‐ and power‐related measures, together with anthropometry in elite golfers.

In male golfers, CHS has consistently shown strong associations with explosive lower body performance. In a recent meta‐analysis by Brennan et al. [9], the strongest pooled associations with CHS were observed for vertical jump impulse (*r* = 0.82). Furthermore, strong associations were also evident with jump peak power (*r* = 0.66), upper body explosive strength (*r* = 0.67)—primarily measured from medicine ball throws for distance, and with maximal vertical countermovement jump (CMJ) height (*r* = 0.53). When considering maximal strength, both lower‐ and upper‐body measures were moderately correlated with CHS (*r* = 0.47–0.48). Anthropometry (pooled across body height, body mass, and arm length) was associated with CHS (*r* = 0.41), whereas flexibility showed no association (*r* = −0.04) [9]. Individual cross‐sectional studies have confirmed the presence of strong‐to‐very strong associations between CHS and CMJ impulse and peak power in elite and highly skilled male golfers (*r* = 0.52–0.81; [10, 11, 12, 13, 14]). Beyond these aforementioned physical measures, Johansen et al. [11] reported very strong associations between CHS and trunk rotational peak power (*r* = 0.81) and isometric bench press (IBP) peak force (*r* = 0.77), respectively. When viewed as a collective body of evidence, it appears that both maximal and ballistic force production measures are most strongly (and consistently) associated with CHS; thus, these parameters may represent important physical attributes that should be targeted for routine testing and monitoring purposes in male golfers.

Evidence in female golfers remains limited, with only a few studies identified in previous systematic reviews [15, 16]. Likewise, a recent meta‐analysis focusing solely on female golfers reported that only three correlational studies met their inclusion criteria, revealing positive correlations between CHS and lower‐body power (*r* = 0.60), upper‐body power (*r* = 0.56–0.57), and measures of maximal strength (*r* = 0.54) as the most prominent associations [17]. In contrast, anthropometric measures showed only moderate associations with CHS (*r* = 0.43), while flexibility demonstrated strong associations (*r* = 0.52–0.71) in contrast to the lack of such relationships in male golfers, which may simply reflect the limited evidence available for comparison. Recently, Robinson et al. [18] examined associations between a comprehensive physical testing battery and golf shot performance in female amateur golfers (handicap ≤ 5). CHS was strongly associated with CMJ‐derived power and impulse measures (*r* = 0.54–0.61), moderately associated with upper‐body pressing strength (*r* = 0.48) and isometric mid‐thigh pull (IMTP) peak force (*r* = 0.38), while CMJ height showed no meaningful association (*r* = −0.11). Collectively, these findings indicate that both maximal and rapid force production capacities are the most relevant physical attributes for CHS in female golfers, despite the limited available evidence.

Although current evidence is growing, most available data come from sub‐elite or mixed‐level cohorts, whereas there is a distinct lack of data in female and true elite‐level golfers. Well‐documented sex differences exist in muscle mass and strength, where males typically demonstrate 30%–50% greater absolute force capacities [19, 20], as well as in driving distance, where males demonstrate ~20%–25% faster CHS on professional tours (PGA Tour average ~115 mph vs. LPGA Tour average ~96 mph; [21]). These differences suggest that the physical determinants of CHS may not apply equally to male and female golfers at the highest performance level.

The present study, therefore, aimed to examine associations between driver club head speed (dCHS) and maximal isometric strength (IMTP, IBP), explosive lower body performance (CMJ height, impulse, peak power, and peak force), trunk rotation peak power, and anthropometry in elite male and female golfers, including PGA and LPGA Tour professionals. Based on prior literature, it was hypothesized that dCHS would show strong associations with trunk rotation peak power, CMJ impulse and peak power, and maximal force capacities in both sexes, albeit with different relative contributions between male and female golfers. Furthermore, analyzing these associations in elite male and female golfers was expected to provide an opportunity to identify potential sex‐specific patterns in how mechanical muscle function relates to driving performance in elite level golfers.

## 2 Materials and Methods

### 2.1 Participants

Forty‐one elite golfers (19 females, 22 males) affiliated with the Danish national golf team volunteered to participate in the study (Table 1). All participants had a minimum of one year of structured strength training experience, were familiar with the present strength testing procedures, and were free from musculoskeletal injuries at the time of testing. Participants ranged from PGA and LPGA Tour professionals to golfers competing on the Access Tour (a pathway tour for professional golfers aiming to reach the highest level). Written informed consent was obtained from all participants, and the study was approved by the local ethical committee (S‐20230062) while adhering to the Declaration of Helsinki.

**TABLE 1:** Baseline descriptive characteristics of the present cohort of elite male (*n* = 22) and female (*n* = 19) golfers.

Variable	Males (mean ± SD)	Males lower dCHS (mean ± SD)	Males higher dCHS (Mean ± SD)	Males Δ%	Females (mean ± SD)	Females lower dCHS (mean ± SD)	Females higher dCHS (mean ± SD)	Δ% (females)	Δ% males versus females
Age (years)	22.2 ± 4.6	21.5 ± 4.8	22.8 ± 4.6	—	21.9 ± 2.7	21.1 ± 1.1	22.7 ± 3.7	—	—
Body height (cm)	183.7 ± 7.2	183.1 ± 6	184.2 ± 7.9	0.6%	167.6 ± 6.1	164.7 ± 5.5	170.8 ± 5.5	3.7%*	9.6%**
Body mass (kg)	79.1 ± 10.2	78.1 ± 9.5	80 ± 10.9	2.4%	64.4 ± 6.0	61.2 ± 6.6	67.6 ± 4.7	10.5%*	22.9%**
Arm length (cm)	78.4 ± 2.8	78.7 ± 3	78.1 ± 2.8	0.8%	71.8 ± 3.1	70.3 ± 2.4	73.3 ± 3.2	4.2%*	9.2%**
Driver CHS (km/h)	185 ± 8.1	178 ± 3.6	190.7 ± 7.2	7.1%**	155 ± 5.5	149.6 ± 3.8	160.2 ± 4.3	7.1%**	19.4%**

*Note:* Values are presented as group mean ± SD for the whole sample, and separately for low‐ and high‐driver club head speed (dCHS) subgroups (median‐split). Δ% indicates percentage difference between low‐ and high‐dCHS groups within each sex, and between males and females overall. — = not calculated.

* *p* < 0.05.

** *p* < 0.001.

### 2.2 Sample Size Estimation and Justification

This trial was an exploratory cross‐sectional study in an elite golfer population. No a priori sample size calculation was performed; the sample size (*n* = 41) was determined by player availability, reflecting the limited accessibility of elite golfers and aligning with previous studies in this field.

### 2.3 Study Overview

All tests were conducted in a single standardized session at the Danish National Team training facilities. Participants were instructed to arrive fully hydrated, to follow their habitual diet in the 48 h prior to testing, and to refrain from any strenuous exercise during the 48 h preceding the test session. Test procedures largely followed those outlined by Johansen et al. [11], with a few protocol modifications (described below).

### 2.4 Experimental Procedures

#### 2.4.1 Anthropometry

Anthropometric measures included body mass, body height, and arm length. Body mass was recorded to the nearest 0.1 kg using a calibrated digital scale, and body height to the nearest 0.5 cm using a stadiometer (SECA 213, Hamburg, Germany). Arm length was measured from the lateral aspect of the acromion to the most distal point of the third finger, with the shoulder at 90° flexion and the elbow fully extended. The mean value of the left and right arms was used for analysis [11].

#### 2.4.2 Golf Swing Protocol

Before testing golf swing parameters, participants completed a standardized warm‐up consisting of 5 min of ergometer cycling at 100 W, followed by dynamic upper‐ and lower body mobility exercises (i.e., wall slides, page turns, arm swings, leg crossovers, leg swings, clock lunges, and overhead squats), light resistance band push/pull drills, glute activation, 3–5 plyometric jumps, and a self‐selected number of warm‐up shots with progressively increasing CHS [11, 13, 22]. After the warm‐up, participants performed 10 maximal golf swings with both their own custom fitted six‐iron and their own driver using unused Titleist Pro V1x 2024 golf balls (Acushnet Company, Fairhaven, MA, USA). Golf shots were executed into a Spornia SPG‐7 practice net (Spornia Inc., Gardena, CA, USA) and recorded using a TrackMan 4 launch monitor (TrackMan A/S, Vedbæk, Denmark). The TrackMan unit was configured according to the manufacturer's recommendations, with the alignment target specified and set to “indoor mode”. Golfers were instructed to hit the ball as forcefully as possible while maintaining their normal swing technique. For further analysis, the average CHS of the three longest shots (total distance) was calculated for each club condition [23].

#### 2.4.3 Countermovement Jumping

Participants performed 3–5 submaximal familiarization jumps. They then completed five maximal CMJs with hands placed on the hips, separated by 60 s rest, and were instructed to jump as high as possible. Jumps were performed on dual force plates (Bertec Corporation, Columbus, OH, USA), which were used to record vertical ground reaction force at 3000 Hz A/D sampling rate, with all output variables calculated in accordance with standardized myoFORCE analysis protocols [24]. For specific analysis, the jump with the greatest flight height was selected [25, 26]. Jump height was calculated from net impulse‐derived take‐off velocity (*h* = *v* ^2^/2*g*). Net impulse was calculated as the time integral of vertical ground reaction force minus body weight during the propulsive (ascending) takeoff phase. Peak takeoff force was defined as the maximal instantaneous vertical ground reaction force generated during the propulsive phase. Peak power was calculated as the maximal instantaneous product of vertical ground reaction force and body center‐of‐mass velocity during the ascending takeoff phase [24, 26, 27].

#### 2.4.4 Isometric Mid‐Thigh Pull, Isometric Bench Press

For both isometric strength tests, participants completed three progressive warm‐up trials at ~50%, 70%, and 90% of their perceived maximal effort. Each maximal trial lasted 5 s, was separated by 2 min rest, and was performed with strong verbal encouragement.

For the isometric mid‐thigh pull (IMTP) test, participants were positioned on a single force platform (specifications listed below) with the bar positioned at the midpoint of the thigh. Knee and hip joint angles were self‐selected within a functional range while ensuring an upright torso, consistent with available protocols [28, 29]. Participants were instructed to “pull as hard and fast as possible.”

For the isometric bench press (IBP) test, participants lay supine on the force platform with knees and hips flexed to avoid external ground contact. The bar was fixed at ~50% of individual arm length above the sternum, yielding an elbow angle of ~70°–80° [30, 31]. Participants were instructed to “press as hard and fast as possible.”

Both tests were performed using a Kairos Strength testing rig (Kairos Strength Inc., Austin, TX, USA) mounted on an AMTI force platform (464 × 508 × 82 mm; Advanced Mechanical Technology Inc., Watertown, MA, USA), with force signals sampled at 3000 Hz and processed in myoFORCE software (Noraxon, USA) without additional filtering. Peak force (N) was determined as the maximal instantaneous vertical ground reaction force, and for each participant, the trial producing the highest peak force was selected for further analysis.

#### 2.4.5 Trunk Rotation Power Test

As described in detail elsewhere [11], participants first completed three progressive warm‐up trials in the cable‐based trunk rotation test at ~50%, ~70%, and ~90% of perceived maximal effort. They then performed 10 maximal trials against a fixed external load (35 kg for males, 25 kg for females), each separated by 15 s of rest. The use of fixed, absolute loads was chosen to ensure standardization across golfers and comparability of results, as previous research has demonstrated high reliability across a spectrum of absolute loads for this test [32]. During all maximal trials, the participants were instructed to rotate as hard and as fast as possible from a simulated six‐iron backswing posture to a follow‐through finishing approximately at the 4 o'clock position [11]. The test was performed using a cable stack device fitted with a linear encoder (T‐Force System, Ergotech Consulting, Murcia, Spain) attached to a custom‐made handle resembling a golf club, with all encoder signals recorded at 1000 Hz A/D sampling rate. Strong verbal encouragement was provided throughout the tests. The outcome variable was peak power (W), defined as the maximal instantaneous product of cable force and stack velocity. Trials were discarded if participants were unable to control the eccentric return phase [32]. For each participant, the trial producing the highest peak power was selected for analysis.

### 2.5 Statistical Analysis

All statistical analyses were performed in Microsoft Excel (Microsoft Corp., Redmond, WA, USA) using the built‐in Analysis Toolpak and custom scripts. Statistical significance was accepted at *p* ≤ 0.05 (two‐tailed). Full sets of TrackMan data were collected for both six‐iron and driver swings, including CHS, ball release speed, carry distance, and total distance. Preliminary analyses revealed very strong to nearly perfect associations between dCHS and these additional golf performance variables, which is in line with previous reports [23]. Based on these findings, dCHS was selected as the primary single outcome variable in the final analysis.

Intrasession test–retest reliability was examined for all test variables based on the three best trials obtained in each participant. Reliability was quantified by within‐subject coefficients of variation (CV_w‐s_, %) [27] and intraclass correlation coefficients (ICC) with 95% confidence intervals (CI) (Table 2). All data were checked for normality using Shapiro–Wilk tests and Q–Q plots, revealing normal (Gaussian) distribution for all variables. Consequently, Pearson's product–moment correlation coefficients (*r*) with 95% confidence intervals and corresponding *p*‐values were calculated to examine associations between dCHS and all performance variables and anthropometric variables obtained, respectively. The strength of correlations was interpreted as weak (*r* < 0.30), moderate (0.30–0.49), strong (0.50–0.69), very strong (0.70–0.89), nearly perfect (0.90–0.99), or perfect (1.0) [11, 33]. For subgroup analyses, male and female golfers were divided separately into high versus low dCHS groups based on a median‐split (upper 50% vs. lower 50%) of their individual dCHS values. Group differences were evaluated using independent‐samples *t*‐testing, with effect sizes expressed as Hedges' *g* and corresponding 95% CI. Because dichotomizing continuous data can reduce statistical power and cause increased within‐group variation, the present subgroup comparisons were included primarily to provide descriptive, practitioner‐oriented insights rather than for primary hypothesis testing.

**TABLE 2:** Performance outcomes and intrasession reliability of countermovement jump (CMJ), isometric mid‐thigh pull, isometric bench press, and trunk rotation power in elite male and female golfers.

Variable	Males mean ± SD	ICC (95% CI) (males)	CV% (males)	Females—mean ± SD	ICC (95% CI) (females)	CV% (females)
Countermovement jump height (cm)	38.6 ± 6.7	0.99 [0.98–0.99]	1.8	28.5 ± 3.3	0.98 [0.95–0.99]	1.9
Countermovement jump peak power (W)	4200 ± 570	0.99 [0.98–0.99]	1.5	2790 ± 320	0.88 [0.76–0.95]	4.9
Countermovement jump net impulse (Ns)	220 ± 33	0.90 [0.81–0.95]	4.1	155 ± 14	0.99 [0.988–0.99]	0.8
Countermovement jump peak force (N)	2075 ± 315	0.99 [0.987–0.997]	2.1	1470 ± 135	0.95 [0.89–0.98]	2.4
Isometric mid‐thigh pull peak force (N)	3160 ± 460	0.94 [0.89–0.97]	3.4	2160 ± 270	0.93 [0.86–0.97]	3.6
Isometric bench press peak force (N)	1740 ± 240	0.98 [0.96–0.99]	1.9	1260 ± 115	0.97 [0.94–0.99]	1.7
Trunk rotation peak power (W)	700 ± 160	0.99 [0.98–0.99]	2.1	382 ± 62	0.99 [0.97–0.99]	2.2

*Note:* Values are presented as mean ± SD together with intraclass correlation coefficient (ICC [95% CI]) and coefficient of variation (CV%).

## 3 Results

**FIGURE 1:**
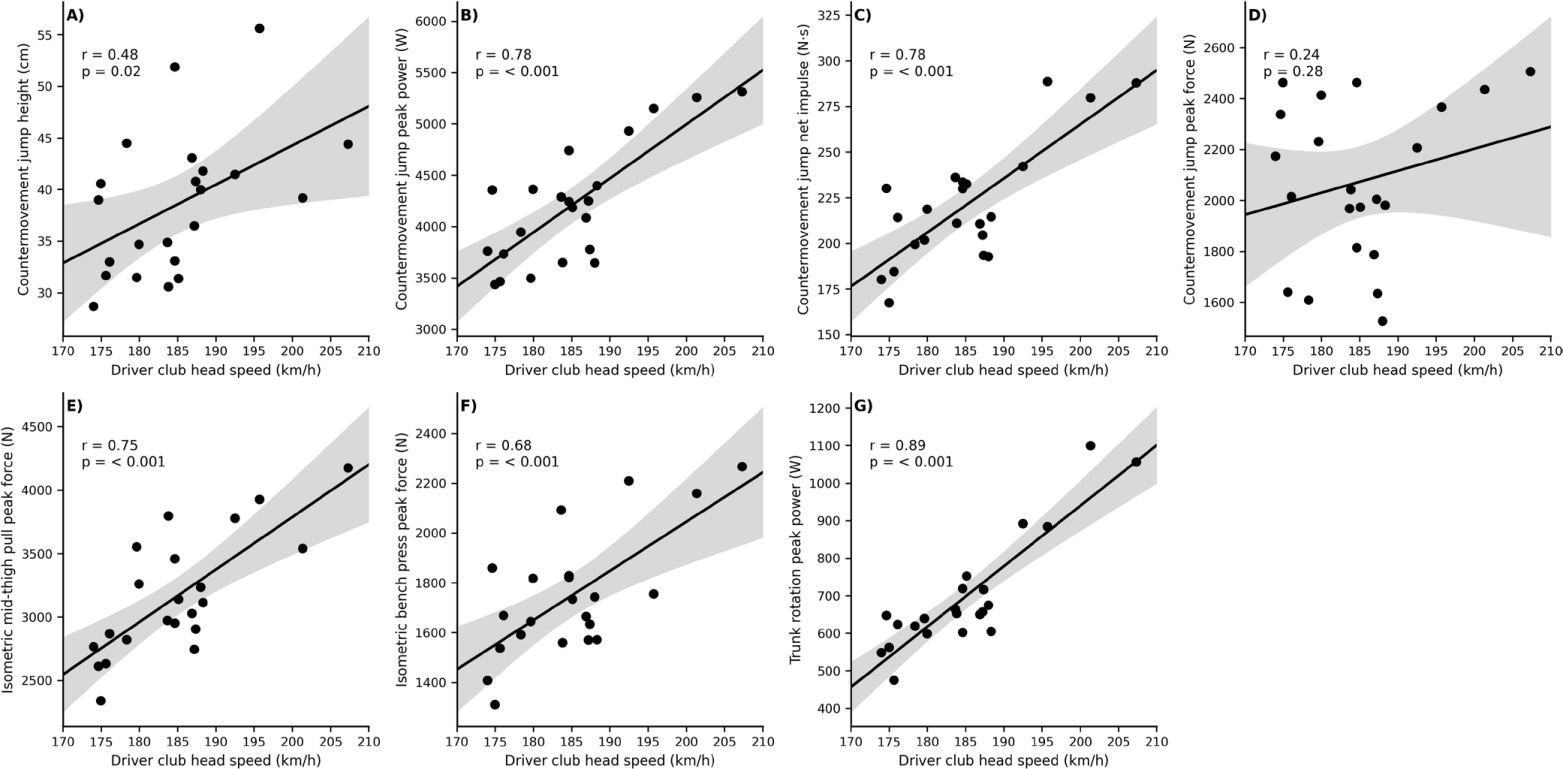
Scatterplots illustrating correlations between driver club head speed and physical performance measures in elite male golfers. Panels show associations with (A) countermovement jump height, (B) countermovement jump peak power, (C) countermovement jump net impulse, (D) countermovement jump peak force, (E) isometric mid‐thigh pull peak force, (F) isometric bench press peak force, and (G) trunk rotation peak power. Solid lines represent linear regression fits, with shaded areas indicating 95% confidence intervals.

In male golfers, dCHS correlated very strongly with trunk rotation peak power (*r* = 0.89 [0.72; 0.96], *p* < 0.001), CMJ impulse (*r* = 0.78 [0.53; 0.90], *p* < 0.001), CMJ peak power (*r* = 0.78 [0.53; 0.90], *p* < 0.001), and IMTP peak force (*r* = 0.75 [0.47; 0.90], *p* < 0.001) (Figure 1). Moreover, IBP peak force was strongly associated with dCHS (*r* = 0.68 [0.35; 0.86], *p* < 0.001). Among anthropometric variables, body mass correlated moderately with dCHS (*r* = 0.45 [0.04; 0.73], *p* = 0.034), while body height (*r* = 0.23 [−0.21; 0.59]) and arm length (*r* = −0.04 [−0.46; 0.39]) showed no significant associations.

**FIGURE 2:**
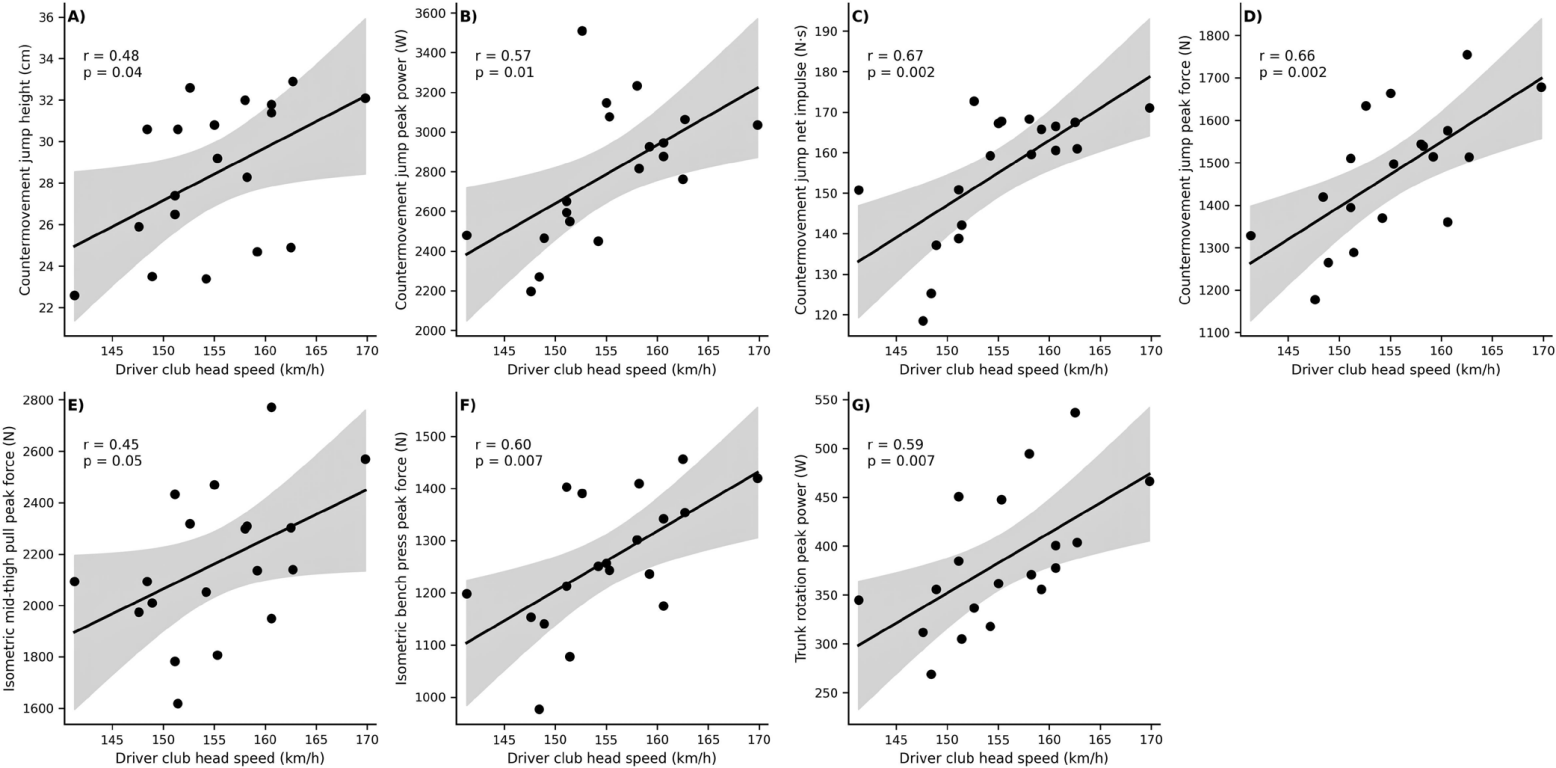
Scatterplots illustrating correlations between driver club head speed and physical performance measures in elite female golfers. Panels show associations with (A) countermovement jump height, (B) countermovement jump peak power, (C) countermovement jump net impulse, (D) countermovement jump peak force, (E) isometric mid‐thigh pull peak force, (F) isometric bench press peak force, and (G) trunk rotation peak power. Solid lines represent linear regression fits, with shaded areas indicating 95% confidence intervals.

In females, dCHS correlated strongly with CMJ impulse (*r* = 0.67 [0.30; 0.87], *p* = 0.002), CMJ peak force (*r* = 0.66 [0.28; 0.86], *p* = 0.002), CMJ peak power (*r* = 0.57 [0.13; 0.81], *p* = 0.011), and IBP peak force (*r* = 0.60 [0.18; 0.83], *p* = 0.007), while trunk rotation peak power also showed a strong association (*r* = 0.59 [0.16; 0.82], *p* = 0.007). Significant correlations were also observed between anthropometry and dCHS in female golfers, with significant correlations for body height (*r* = 0.51 [0.07; 0.78], *p* = 0.027) and body mass (*r* = 0.49 [0.05; 0.77], *p* = 0.033), while arm length showed a non‐significant moderate association (*r* = 0.39 [−0.08; 0.72], *p* = 0.095) (Figure 2).

**FIGURE 3:**
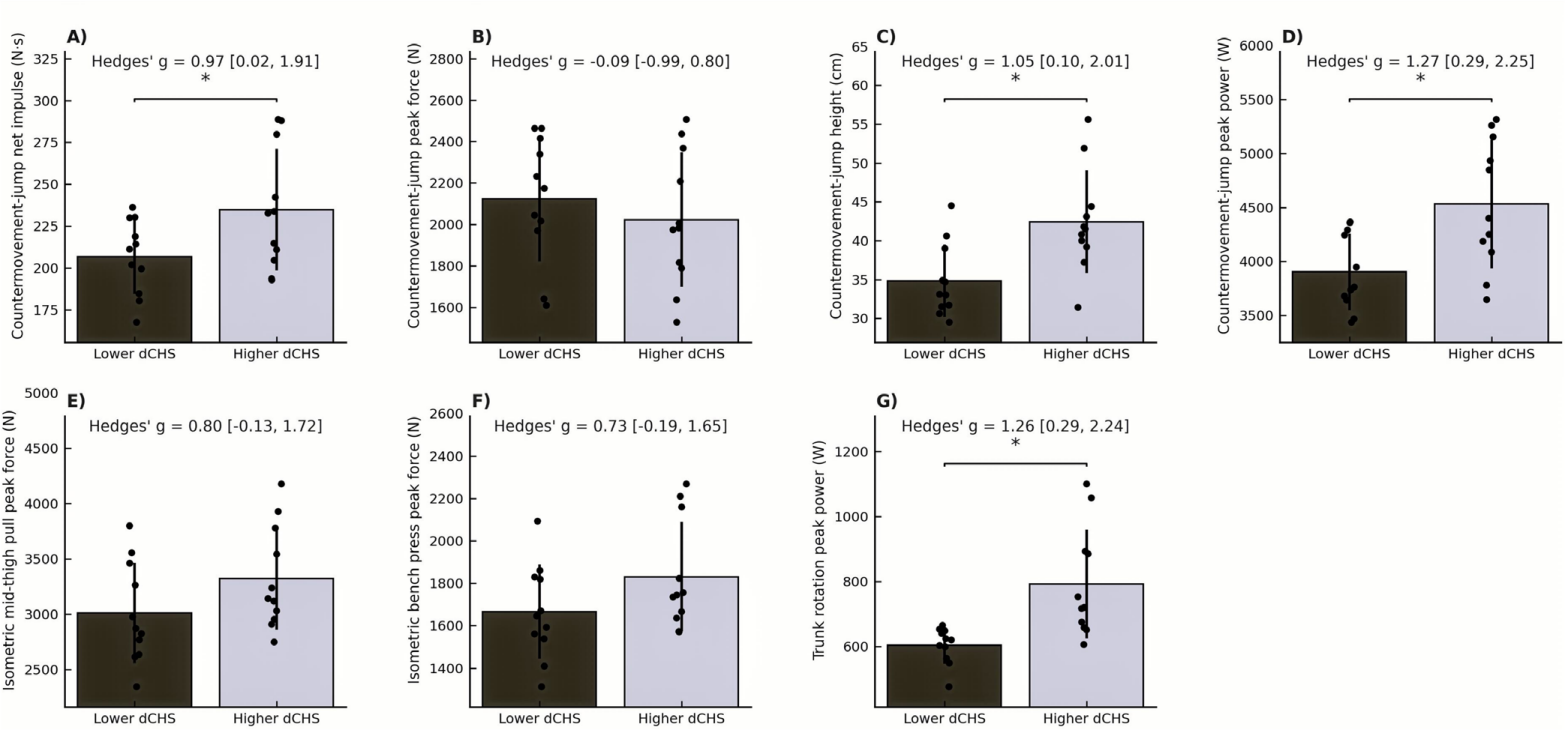
Median‐split comparisons of higher‐ vs. lower driver club head speed groups in elite male golfers. Panels show differences in (A) countermovement jump net impulse, (B) countermovement jump peak force, (C) countermovement jump height, (D) countermovement jump peak power, (E) isometric mid‐thigh pull peak force, (F) isometric bench press peak force, and (G) trunk rotation peak power. Bars represent group means with individual data points overlaid. Asterisks indicate significant between‐group differences (*p < 0.05). Effect sizes are reported as Hedges' g with 95% confidence intervals.

Median‐split analyses confirmed that high‐dCHS males outperformed low‐dCHS males in CMJ peak power (+16.8%, *p* = 0.006, *g* = 1.27), CMJ impulse (+14.6%, *p* = 0.03, *g* = 0.97), and trunk rotation peak power (+28.6%, *p* = 0.006, *g* = 1.26) (Figure 3).

**FIGURE 4:**
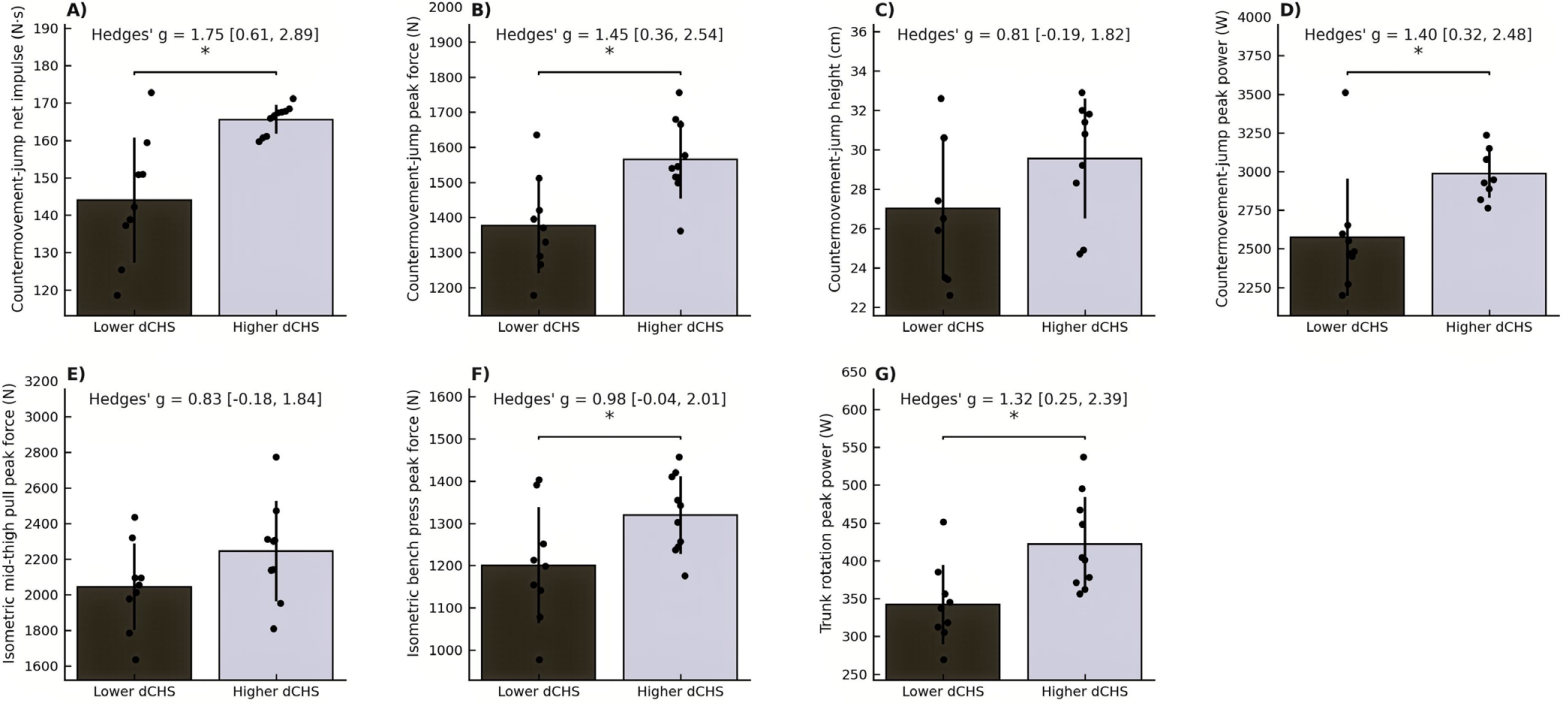
Median‐split comparisons of higher‐ vs. lower driver club head speed groups in elite female golfers. Panels show differences in (A) countermovement jump net impulse, (B) countermovement jump peak force, (C) countermovement jump height, (D) countermovement jump peak power, (E) isometric mid‐thigh pull peak force, (F) isometric bench press peak force, and (G) trunk rotation peak power. Bars represent group means with individual data points overlaid. Asterisks indicate significant between‐group differences (*p < 0.05). Effect sizes are reported as Hedges' g with 95% confidence intervals.

Likewise, high‐dCHS females outperformed low‐dCHS females in CMJ impulse (+15.0%, *p* = 0.001, *g* = 1.75), CMJ peak force (+13.6%, *p* = 0.004, *g* = 1.45), CMJ peak power (+16.1%, *p* = 0.005, g = 1.40), IBP peak force (+9.9%, *p* = 0.04, *g* = 0.98), and trunk rotation peak power (+23.4%, *p* = 0.008, *g* = 1.32) (Figure 4).

## 4 Discussion

The present study examined potential relationships between dCHS and selected measures of physical performance in elite male and female golfers. As a main finding, male golfers demonstrated very strong correlations between dCHS and trunk rotation peak power, CMJ impulse and peak power, and IMTP peak force, respectively, alongside a strong association with IBP peak force. Also representing a main finding, female golfers showed dCHS to be strongly correlated with CMJ impulse, CMJ peak force, trunk rotation peak power, and IBP peak force, while anthropometric variables such as body height and mass were also significantly associated with dCHS in females. Median‐split analyses confirmed that high‐dCHS golfers of both sexes outperformed their lower‐dCHS counterparts across key strength and power measures, with anthropometry additionally distinguishing groups in females but not in males.

In male golfers, CMJ impulse and peak power were very strongly correlated with dCHS (Figure 1). These findings confirm earlier reports in national‐ and elite‐level golfers, where CMJ‐derived impulse and power metrics have consistently been among the strongest predictors of dCHS [10, 11, 12, 13, 14]. A likely explanation for this strong relationship may be the similarity in force–time characteristics between the propulsive phase of the CMJ (≈200–300 ms; [34]) and the golf downswing (≈230–284 ms; [35]), potentially making CMJ derived impulse‐ (in particular) and power‐based measures ecologically valid indicators of CHS performance. For comparison, CMJ jump height showed only a moderate association with dCHS. As a possible explanation, excess body mass may be detrimental to maximal jump height, while during a golf swing it may simultaneously contribute to generating greater momentum (mass · velocity) to the club, which in turn may help to increase the kinetic energy of the club head at the instant of ball impact, hence resulting in increased driving distance [9]. Staying with male golfers, trunk rotation peak power showed the single strongest relationship with dCHS, consistent with findings of Johansen et al. [11], highlighting the trunk's central role in transferring energy through the kinetic chain during the golf swing. Using peak trunk rotation power as a specific outcome variable appears particularly relevant, as it captures both force and velocity, aligning with the explosive nature of the golf swing [36]. IMTP peak force was also found to correlate very strongly with dCHS, in line with previous investigations [11], although weaker or absent associations have also been reported [10]. These conflicting observations between the IMTP and CHS may be because some golfers were tested during competitive periods [10], where they may have avoided maximal‐effort physical tasks in an attempt to ensure full readiness for competition.

In biomechanical terms, the IMTP test remains relevant in golfers, as contraction time to peak force (~256 ms) closely mirrors the time frame of the downswing [37], and because the test engages both the hip and knee extensors that are known to contribute critically to force generation and weight transfer during the downswing [38]. Likewise, IBP peak force was strongly associated with dCHS, aligning with evidence that upper body pressing strength (as assessed by the IBP test) contributes to force transfer during the golf downswing [11, 18]. This is, in part, supported by electromyography data showing the pectoralis major to have a high level of muscle activity during the golf downswing [39]. Finally, body mass correlated moderately with dCHS, whereas body height and arm length showed no associations, suggesting that in male golfers' high levels of maximal and rapid strength and power capacities are more critical for dCHS than large body size alone.

In female golfers, dCHS was strongly associated with CMJ impulse, peak force, and peak power, highlighting the combined importance of explosive and absolute lower body extensor muscle force capacities in this group (Figure 2). A likely explanation for the association between CMJ peak force and dCHS could be that CMJ peak force is positively influenced by body mass [34, 40], which in the present female golfers also was positively correlated with dCHS, underlining a supportive role of body mass in female golfers. In contrast, CMJ jump height showed a moderate correlation with dCHS, supporting earlier observations that jump height–based measures provide limited insight into the explosive physical demands of the golf swing in female golfers [18, 22]. Conversely, trunk rotation peak power was strongly correlated with dCHS in the present female golfers, and although the association was somewhat weaker than observed in male golfers, the present data strongly support the relevance of the trunk rotator musculature in transferring energy through the kinetic chain from the legs to the arms. Underlining the novelty of this observation, to our best knowledge, the present study is the first to demonstrate such a relationship in female elite golfers. Likewise, IBP peak force was strongly associated with dCHS, suggesting that upper body pressing strength contributes meaningfully to dCHS in female golfers, aligning with recent observations obtained in high‐level female golfers [18]. Notably, however, IMTP peak force showed only a moderate association with dCHS, indicating that in female golfers, maximal lower body force capacity may play a lesser role compared with measures of maximal and explosive upper body strength and power.

The present median‐split analyses in the subgroup of male golfers revealed high‐dCHS golfers to have superior levels of CMJ impulse, CMJ peak power, trunk rotation peak power, and CMJ height (Figure 3), respectively. Notably, CMJ height distinguished the high and low dCHS groups despite only moderate correlations being observed with dCHS, suggesting this simple measure (CMJ height) to provide a practical alternative when force plate–based assessments are not available. IMTP and IBP peak force showed no significant group differences between male high and low dCHS golfers, although moderate effect sizes indicated a tendency toward higher capacities in the high‐dCHS group. For female golfers, median‐split analyses showed that high‐dCHS golfers significantly outperformed their lower‐dCHS counterparts in terms of CMJ impulse, CMJ peak force, CMJ power, IBP peak force, and trunk rotation peak power (Figure 4). Body height and body mass also distinguished between the female subgroups, highlighting that greater body height and mass contributed substantially to dCHS differences [17, 18, 22]. By comparison, no significant group difference was observed for IMTP, though a moderate ES indicated a tendency toward higher force capacities in the high‐dCHS group. This pattern suggests that maximal lower body force capacity may be less decisive in female elite golfers. One explanation could potentially be that the IMTP requires substantial upper body stabilization to fully express lower body force, which could constrain performance in females despite adequate lower body capacity. Regardless, the significant group difference between female high and low dCHS performers in IBP peak force underscores the importance of high upper body pressing strength for effective force transfer during the golf swing. This interpretation aligns with kinematic evidence showing that elite female golfers exhibit slower distal segment velocities and longer downswing durations compared to male golfers [41, 42]. Whilst this explanation is anecdotal, this may potentially reflect a reduced efficiency in transferring energy through the kinetic chain, particularly in the transition from the hips through the trunk to the arms, which may partly stem from reduced upper body strength levels. Consequently, designated strength training protocols to increase upper body strength may have particular relevance for increasing dCHS in elite female golfers [43].

Several limitations should be acknowledged in the present study. Firstly, the study was designed as a cross‐sectional study, which limits interpretation of the observed associations to a single time point. Consequently, no causal inferences could be made regarding whether changes in isolated strength or power capacities would translate into faster dCHS in the present cohort. Secondly, although the opportunity to recruit a full sample of elite level golfers may be considered a major strength, the observations may not necessarily generalize to sub‐elite or junior golfers. In addition, only intrasession reliability was assessed. All measures demonstrated excellent reliability within a single testing session. However, inter‐session reliability was not examined and should be considered in future studies. Finally, testing was conducted indoors under sport‐specific conditions outside competition, and the setup may differ from tournament play. Therefore, residual confounding due to technical execution cannot be excluded.

## 5 Perspective

Across males and females, dCHS showed its strongest associations with trunk rotation peak power, CMJ impulse and peak power, and upper body pressing strength (IBP). However, the pattern of correlates differed by sex. In females, anthropometric characteristics and measures of absolute force production (e.g., CMJ peak force and IBP peak force) appeared more closely linked to dCHS, whereas in males, dCHS was more closely associated with indices of mechanical muscle function, such as IMTP peak force together with CMJ impulse and peak power. The median‐split analyses provide a practical illustration of these sex‐specific profiles by showing consistently higher strength and power performance in the higher dCHS groups, with anthropometry contributing more clearly to subgroup separation in females.

These findings align with the temporal and mechanical demands of the golf downswing, which occurs within ~300 ms and requires rapid force production and effective transmission of force and angular momentum through the kinetic chain [35]. Evidence from highly skilled male golfers supports trunk rotation power testing as a monitoring target for rotational power development [11]. From an applied perspective, CMJ, IMTP, IBP, and trunk rotation power testing appear well suited for profiling and longitudinal monitoring in elite golfers, provided results are interpreted separately for males and females. Future studies should determine to which extent targeted training‐induced improvements in these physical capacities may translate into sustained increases in dCHS and competitive performance, as suggested by recent interventions implemented within competitive golf environments [43].

## Funding

This project was supported by the Team Danmark Elite Sports Association through a grant from the Novo Nordisk Foundation (Grant NNF22SA0078293) and by the Danish Ministry of Culture.

## Disclosure

The authors have nothing to report.

## Conflicts of Interest

The authors declare no conflicts of interest.

## Data Availability

The data that support the findings of this study are available from the corresponding author upon reasonable request.
